# Msn2/4 transcription factors positively regulate expression of Atg39 ER-phagy receptor

**DOI:** 10.1038/s41598-021-91480-0

**Published:** 2021-06-07

**Authors:** Tomoaki Mizuno, Kenji Irie

**Affiliations:** Department of Molecular Cell Biology, Faculty of Medicine, University of Tsukuba, 1-1-1 Tennoudai, Tsukuba, 305-8575 Japan

**Keywords:** Autophagy, Cell signalling, Organelles

## Abstract

Selective autophagy requires the autophagy receptor specifically localizing to the target for degradation. In the budding yeast, Atg39 and Atg40 function as an autophagy receptor for the endoplasmic reticulum (ER)-selective autophagy, referred to as ER-phagy. The expression level of the *ATG39* gene is increased in response to ER stress and nitrogen starvation. Under unstressed conditions, *ATG39* transcription is repressed by Mig1/2 repressors. ER stress activates Snf1 AMP-activated protein kinase (AMPK), which negatively regulates Mig1/2 and consequently derepresses *ATG39* transcription. However, *ATG39* expression is still induced by ER stress and nitrogen starvation in the absence of Snf1, suggesting that additional molecules are involved in regulation of *ATG39* expression. Here, we identify Msn2/4 transcription factors as an activator of *ATG39* transcription. Not only *ATG39* promoter activity but also ER-phagy are downregulated by loss of Msn2/4 and disruption of Msn2/4-binding consensus sequences located in the *ATG39* promoter. We also find that the cAMP-dependent protein kinase pathway is involved in Msn2/4-mediated transcriptional regulation of *ATG39*. Our results suggest that yeast ER-phagy is appropriately controlled through modulation of the expression level of the ER-phagy receptor involving multiple signaling pathways and transcription factors.

## Introduction

Autophagy is an evolutionarily conserved process which degrades intracellular components^1,2^. Autophagy is categorized into two types, macroautophagy and microautophagy. In macroautophagy, double-membrane vesicles termed autophagosomes are generated to transport target constituents into degradative organelles (the vacuole in the budding yeast). On the other hand, microautophagy transports the targets without forming autophagosomes. Macroautophagy is further categorized into two types, non-selective macroautophagy and selective macroautophagy, which degrade intracellular components non-specifically and specifically, respectively^3–5^. Selective macroautophagy includes mitophagy, pexophagy, and ER-phagy, which specifically degrade mitochondria, peroxisomes, and the endoplasmic reticulum (ER), respectively.

In selective macroautophagy, the autophagy receptor localizing to the target organelle is required for recruiting the core autophagy-related proteins that function in the autophagosome formation^3–5^. In the budding yeast, Atg39 and Atg40 have been identified as an autophagy receptor specific for ER-phagy^6^. The budding yeast ER consists of two distinct regions, the perinuclear ER and the cortical ER, which are connected by cytoplasmic ER^7^. Atg39 and Atg40 show the distinct localization pattern in cells treated with rapamycin, a compound that mimics nitrogen starvation^6^: Atg39 specifically localizes to the perinuclear ER; Atg40 predominantly localizes to the cortical and cytoplasmic ER. Reflecting their localization pattern, the perinuclear ER is mainly degraded by Atg39 after rapamycin treatment, whereas the cortical and cytoplasmic ER is mainly degraded by Atg40^6^. ER-phagy is also initiated by ER stress, the condition where aberrant proteins accumulate in the ER lumen and membrane^8,9^. However, differently from rapamycin treatment, Atg39 plays a major role in degradation of both the perinuclear ER and the cortical and cytoplasmic ER during ER stress response^9^.

Regulation of the expression levels of the core autophagy-related proteins and the autophagy receptors is crucial for modulating the activity of autophagy^8–14^. The core autophagy-related proteins are induced when cells are cultivated under nitrogen-starved conditions. The mRNA levels of the core autophagy-related genes are controlled at both the transcriptional and post-transcriptional levels^11–14^. Like the core autophagy-related proteins, the expression levels of autophagy receptors are elevated under conditions that induce selective autophagy^6,15,16^. The expression levels of *ATG39* and *ATG40* are upregulated by nitrogen starvation and ER stress^6,8,9^. *ATG40* expression is regulated by the Pho23-Rpd3L histone deacetylase complex^8^. We have previously identified Snf1 AMP-activated protein kinase (AMPK) and two closely related transcriptional repressors, Mig1 and Mig2, as a regulator of *ATG39* expression^9^. Under unstressed conditions, Mig1 and Mig2 redundantly repress *ATG39* promoter activity. ER stress leads to activation of Snf1, which consequently promotes nuclear export of Mig1 and Mig2 to derepress the *ATG39* promoter. However, the existence of additional molecules regulating *ATG39* promoter activity has been predicted from the following observations: ER stress upregulated *ATG39* expression in the *mig1 mig2* double mutant cells; *ATG39* induction in response to nitrogen starvation was normally occurred in the *snf1* mutant cells.

In this study, we identified two closely related transcription factors, Msn2 and Msn4, as a regulator of *ATG39* expression by a genetic approach using the reporter cells. Loss of Msn2 and Msn4 diminished *ATG39* induction caused by ER stress and nitrogen starvation. Activation of the *ATG39* promoter induced by ER stress and nitrogen starvation was significantly inhibited by disruption of Msn2/4-binding consensus sequences located in the *ATG39* promoter. Mutations in the *MSN2* and *MSN4* genes reduced the activity of ER-phagy under ER-stressed and nitrogen-starved conditions. We also found that loss of Pde1/2 cyclic AMP (cAMP) phosphodiesterases downregulates *ATG39* promoter activity and ER-phagy. These results suggest that ER-phagy is positively regulated by Msn2/4 through activation of the *ATG39* promoter and that Msn2/4 themselves are negatively regulated by protein kinase A (PKA).

## Results

### Development of the reporter cell to monitor ATG39 promoter activity

**Figure 1:**
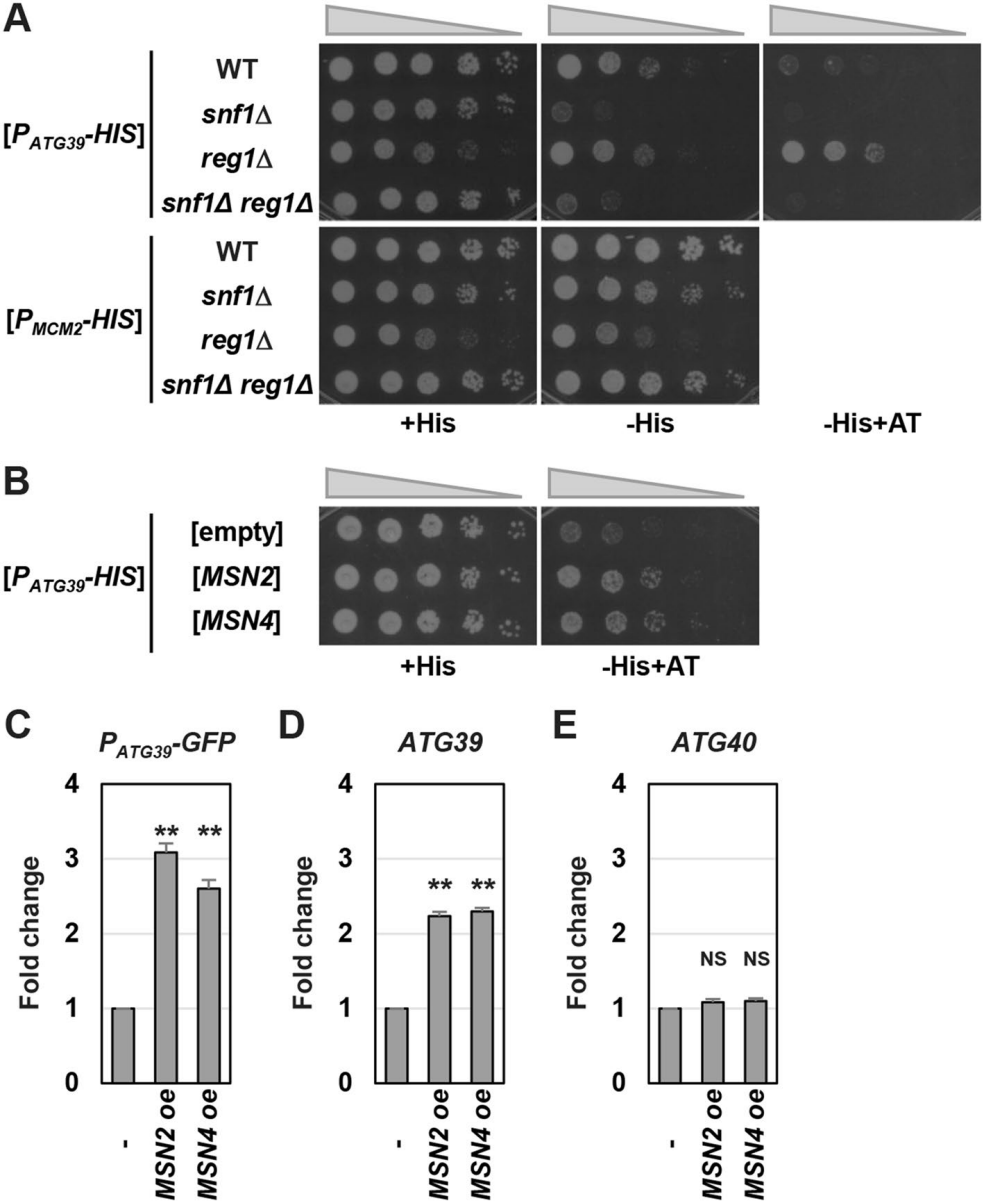
*ATG39* expression is increased by Msn2/4 overexpression. (**A**) Effects of Snf1 activity on growth of cells harboring the *P*_*ATG39*_*-HIS3* reporter. Wild-type (WT) and indicated mutant strains harboring the *P*_*ATG39*_*-HIS3* or *P*_*MCM2*_*-HIS3* reporters were spotted onto synthetic defined media lacking or containing histidine (His) and 3-amino-1*H*-1,2,4-triazole (AT), and incubated at 25 °C. (**B**) Effects of *MSN2*/*4* overexpression on growth of cells harboring the *P*_*ATG39*_*-HIS3* reporter. Wild-type (WT) cells harboring the *P*_*ATG39*_*-HIS3* reporter and the plasmids with or without the *MSN2*/*4* genes were spotted onto synthetic defined media lacking or containing histidine (H) and 3-amino-1H-1,2,4-triazole (AT), and incubated at 25 °C. (**C**) Effects of *MSN2*/*4* overexpression on expression of the *P*_*ATG39*_*-GFP* reporter. Wild-type strains harboring the *P*_*ATG39*_*-GFP* reporter and the plasmids with or without the *MSN2*/*4* genes were grown at 25 °C until exponential phase and then harvested. The *GFP* mRNA levels were quantified by qRT-PCR analysis, and relative mRNA levels were calculated using *ACT1* mRNA. The values are plotted as the fold change from cells harboring the empty plasmids. The data show mean ± SEM (n = 3). **P < 0.01 as determined by Student’s *t*-test. (**D**,**E**) Effects of *MSN2*/*4* overexpression on the mRNA levels of *ATG39* (**D**) and *ATG40* (**E**). Wild-type strains harboring the plasmids with or without the *MSN2*/*4* genes were grown at 25 °C until exponential phase and then harvested. The mRNA levels were quantified by qRT-PCR analysis, and relative mRNA levels were calculated using *ACT1* mRNA. The values are plotted as the fold change from cells harboring the empty plasmids. The data show mean ± SEM (n = 3). **P < 0.01 as determined by Student’s *t*-test. NS, not statistically significant (*P* > 0.05).

We previously showed that the *ATG39* promoter is activated by ER stress and nitrogen starvation^9^. Activation of the *ATG39* promoter caused by ER stress is only partially reduced by *snf1* mutation. Furthermore, the *ATG39* promoter is normally activated by nitrogen starvation in the *snf1* mutant cells. Thus, additional molecules should regulate *ATG39* promoter activity. To measure *ATG39* promoter activity indirectly by monitoring the cell growth and further identify the regulator of *ATG39* transcription genetically, we constructed the *P*_*ATG39*_*-HIS3* reporter which expresses the *HIS3* gene under the control of the *ATG39* promoter. Previously, we found that *ATG39* promoter activity under unstressed conditions is enhanced by *reg1* mutation which causes Snf1 hyperactivation^9,17–19^. Therefore, we first compared growth between wild-type and *reg1* mutant cells when *P*_*ATG39*_*-HIS3* was provided as a sole functional *HIS3* gene. Compared with wild-type cells, *reg1* mutant cells grew slowly on synthetic defined media containing histidine (SD + His) (Fig. 1A). Nevertheless, *reg1* mutant cells could grow on synthetic defined media lacking histidine (SD-His) at a level comparable to wild-type cells. Furthermore, *reg1* mutant cells grew better than wild-type cells in the presence of 3-amino-1*H*-1,2,4-triazole (AT), a competitor of the *HIS3* gene product. Increased growth observed in *reg1* mutant cells was suppressed by *snf1* mutation, indicating that this *reg1* phenotype is caused by hyperactivation of Snf1. We performed similar experiments using the *P*_*MCM2*_*-HIS3* construct which expresses the *HIS3* gene under the control of the *MCM2* promoter. The promoter activity of *MCM2* under unstressed conditions was relatively higher than that of *ATG39* and was unaffected by *reg1* mutation (Supplementary Fig. 1). Consistent with this, histidine depletion had little effects on the growth of wild-type and *reg1* mutant cells harboring *P*_*MCM2*_*-HIS3* (Fig. 1A). These results indicate that the *P*_*ATG39*_*-HIS3* reporter can be utilized in monitoring *ATG39* promoter activity.

### Msn2/4 overexpression facilitates ATG39 promoter activity

We screened a multicopy genomic library to identify genes whose overexpression facilitates growth of cells harboring *P*_*ATG39*_*-HIS3* on synthetic defined media lacking histidine and containing AT (SD-His + AT). We isolated 16 plasmids which facilitate cell growth on SD-His + AT and were categorized into 4 groups (Supplementary Table 1). The group I was comprised of 8 plasmids that carry genomic regions including the *ATR1* (AminoTriazole Resistance 1) gene^20^, suggesting that these plasmids confer AT resistance without upregulating *HIS3* expression. The group II included 4 plasmids, which contain the *MSN2* gene encoding a transcription factor^18,21^. The groups III and IV were comprised of 3 and 1 plasmids, respectively, and they carried a gene known to encode a negative regulator of the RAS/protein kinase A (PKA) pathway, *PDE2* or *GPB1*^18,22,23^. PKA negatively regulates Msn2 through phosphorylation^18,24,25^. Therefore, we hypothesized that Msn2 overexpression upregulates *ATG39* transcription and that overexpression of Pde2 and Gpb1 activates Msn2 by downregulating the PKA activity. To examine whether Msn2 overexpression really increases growth of cells harboring *P*_*ATG39*_*-HIS3* on SD-His + AT, we constructed a multicopy plasmid expressing Msn2 alone. Overexpression of Msn2 did not change the growth rate of the reporter cell on SD + His but increased it on SD-His + AT (Fig. 1B). The budding yeast has a gene closely related to *MSN2*, *MSN4*^18,21^. Therefore, we tested whether Msn4 is also involved in *ATG39* expression. Msn4 overexpression facilitated growth of the reporter cell on SD-His + AT, but not on SD + His (Fig. 1B).

We next investigated whether the mRNA expressed from the *ATG39* promoter is increased by Msn2/4 overexpression using the *P*_*ATG39*_*-GFP* reporter which expresses the *GFP* gene under the control of the *ATG39* promoter^9^. The quantitative real-time RT-PCR (qRT-PCR) analysis showed that *GFP* expression from the *P*_*ATG39*_*-GFP* reporter was enhanced by Msn2/4 overexpression (Fig. 1C). We next examined the effect of Msn2/4 overexpression on the expression levels of endogenous genes. *ATG39* expression was upregulated by Msn2/4 overexpression (Fig. 1D). However, Msn2/4 overexpression had no effect on the expression level of *ATG40*, another gene encoding an ER-phagy receptor (Fig. 1E). These results suggest that Msn2/4 overexpression specifically increases *ATG39* transcription.

### Msn2/4 activate ATG39 promoter activity during ER stress response and nitrogen starvation

**Figure 2:**
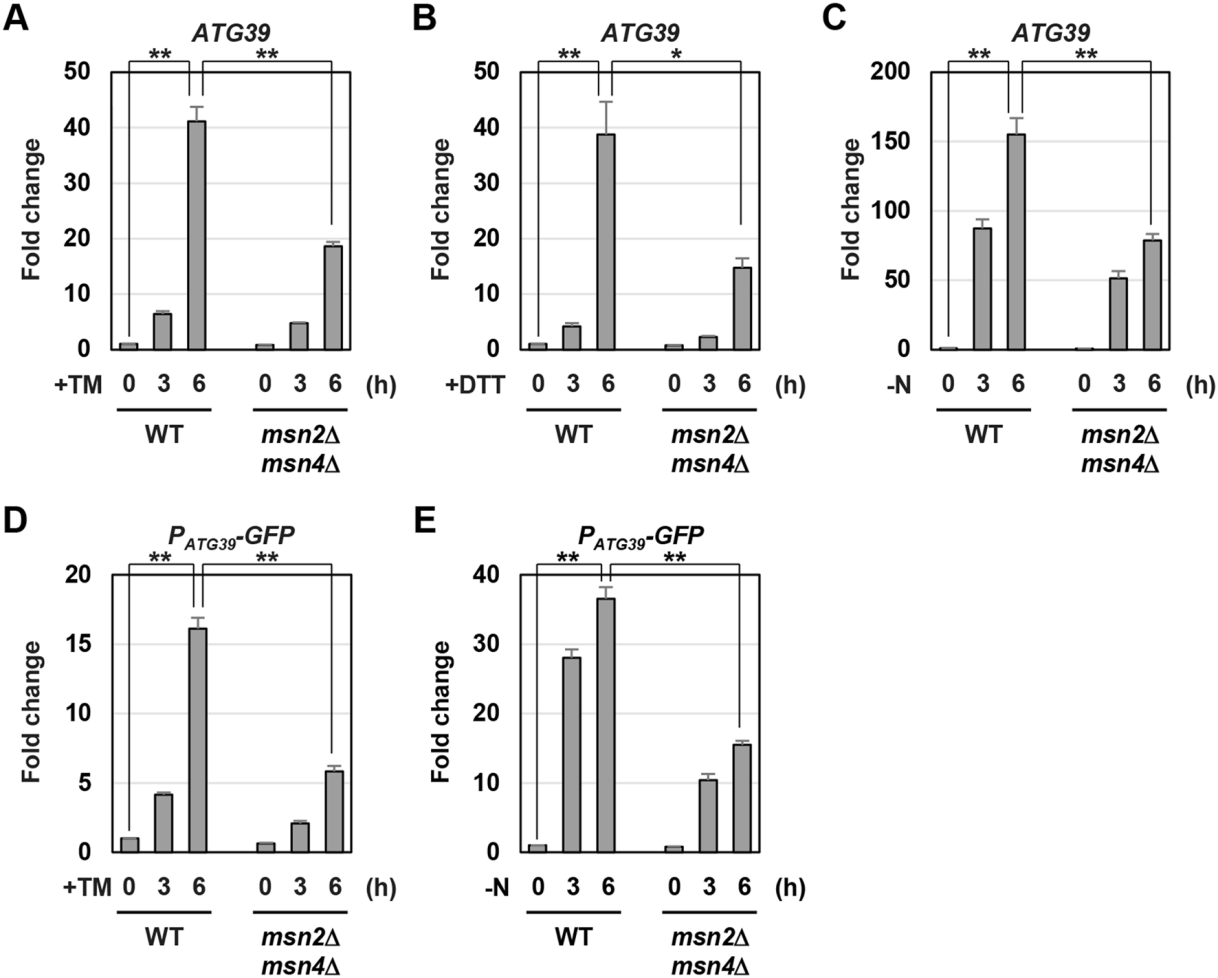
*ATG39* expression is reduced by *msn2 msn4* mutations. (**A**–**C**) The *ATG39* mRNA levels in *msn2 msn4* mutant. Wild-type (WT) and *msn2 msn4* mutant strains were grown at 25 °C until exponential phase and treated with 3 μg/ml tunicamycin (TM) (**A**) or 6 mM dithiothreitol (DTT) (**B**) or incubated under nitrogen-starved conditions (**C**) for the indicated time. The *ATG39* mRNA levels were quantified by qRT-PCR analysis, and relative mRNA levels were calculated using *ACT1* mRNA. The values are plotted as the fold change from wild-type cells at the time of ER stressors addition or nitrogen removal. The data show mean ± SEM (n > 4). *P < 0.05 and **P < 0.01 as determined by Student’s *t*-test. (**D**,**E**) Expression of *P*_*ATG39*_*-GFP* reporter in *msn2 msn4* mutant. Wild-type (WT) and indicated mutant strains were grown at 25 °C until exponential phase and treated with 3 μg/ml tunicamycin (TM) (**D**) or incubated under nitrogen-starved conditions (**E**) for the indicated time. The *ATG39* mRNA levels were quantified by qRT-PCR analysis, and relative mRNA levels were calculated using *ACT1* mRNA. The values are plotted as the fold change from wild-type cells at the time of TM addition or nitrogen removal. The data show mean ± SEM (n > 3). **P < 0.01 as determined by Student’s *t*-test.

*ATG39* expression is induced by ER stress and nitrogen starvation^6,9^. We therefore asked if Msn2/4 are involved in *ATG39* induction caused by ER stress. In wild-type cells, *ATG39* mRNA was markedly increased by treatment with tunicamycin, which causes ER stress by inhibiting N-linked glycosylation (Fig. 2A). *ATG39* induction was diminished in the *msn2 msn4* double mutant cells, although it was normally occurred in the *msn2* and *msn4* single mutant cells (Fig. 2A and Supplementary Fig. 2A). We also found that *msn2 msn4* double mutations reduced *ATG39* induction caused by dithiothreitol (DTT), which causes ER stress by inhibiting the disulfide bond formation (Fig. 2B). We next examined whether Msn2/4 are involved in *ATG39* induction caused by nitrogen starvation. *ATG39* induction was diminished in the *msn2* and *msn4* single mutant cells (Supplementary Fig. 2B). The *msn2* and *msn4* mutations synergistically reduced *ATG39* induction (Fig. 2C). We also measured *ATG40* mRNA levels and found that *ATG40* induction in response to ER stress and nitrogen starvation was unaffected by *msn2 msn4* double mutations (Supplementary Fig. 3). These results suggest that Msn2/4 are specifically involved in *ATG39* induction.

ER stress and nitrogen starvation enhance *ATG39* promoter activity^9^. Therefore, we examined whether Msn2/4 are involved in activation of the *ATG39* promoter in response to ER stress and nitrogen starvation. *GFP* expression from the *P*_*ATG39*_*-GFP* reporter was increased by ER stress and nitrogen starvation; however, their induction was significantly diminished by *msn2 msn4* double mutations (Fig. 2D,E). These results suggest that during ER stress response and nitrogen starvation, Msn2/4 positively regulate *ATG39* expression via its promoter.

### Msn2/4 regulate ATG39 promoter activity via the STRE elements

**Figure 3:**
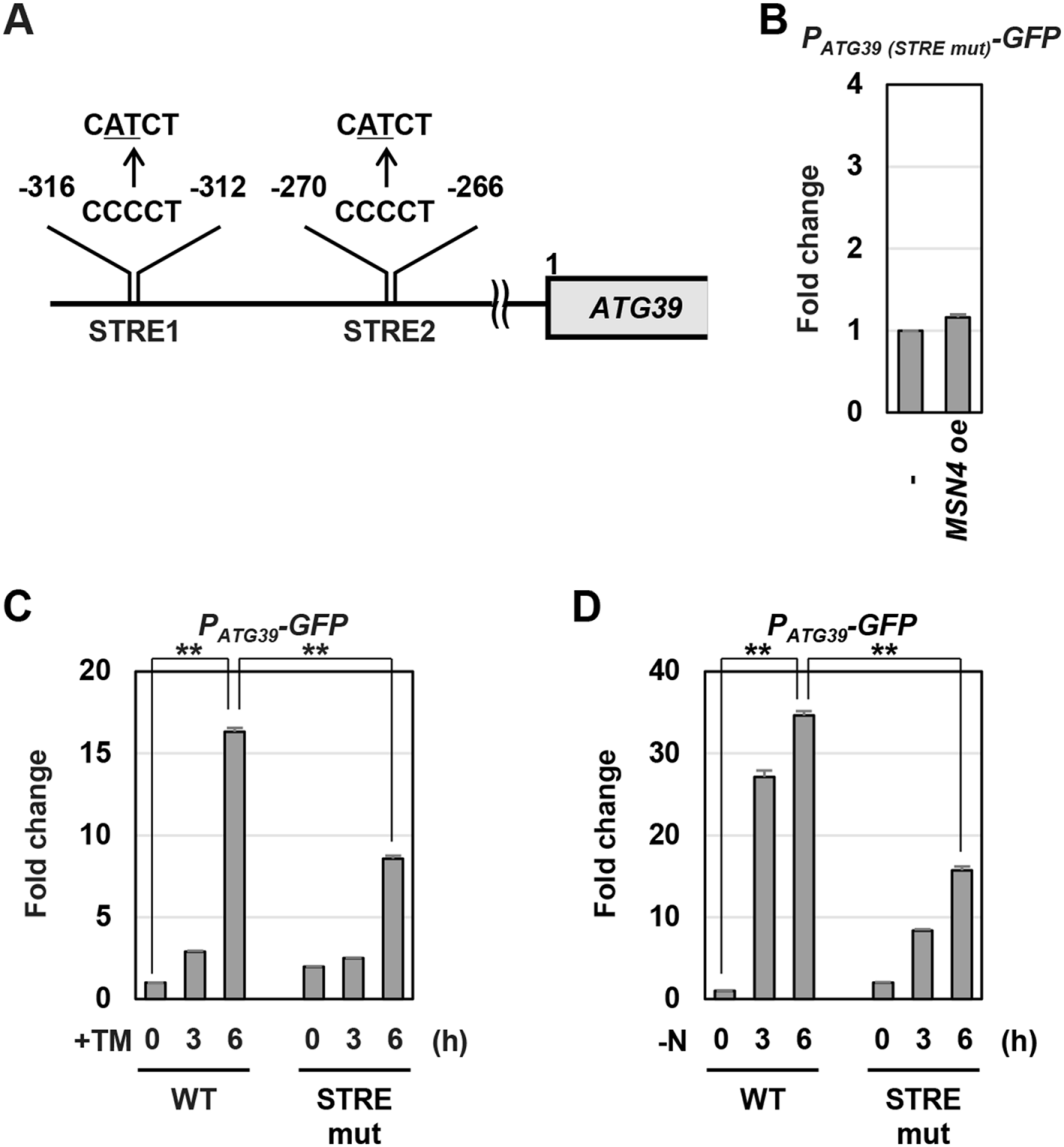
Msn2/4 positively regulate *ATG39* promoter activity through the STRE elements. (**A**) Two putative STRE elements in *ATG39* promoter region. (**B**) Effects of *MSN4* overexpression on expression of the *P*_*ATG39*_*-GFP* reporter mutated in putative STRE elements. Wild-type (WT) strains harboring the *P*_*ATG39*_*-GFP* reporter mutated in putative STRE elements and the plasmids with or without the *MSN4* gene were grown at 25 °C until exponential phase, and then harvested. The *GFP* mRNA levels were quantified by qRT-PCR analysis, and relative mRNA levels were calculated using *ACT1* mRNA. The values are plotted as the fold change from cells harboring the plasmids without the *MSN4* gene. The data show mean ± SEM (n = 4). NS, not statistically significant (*P* > 0.05), as determined by Student’s *t*-test. (**C**,**D**) Effects of mutations in putative STRE elements on *ATG39* upregulation induced by ER stress and nitrogen starvation. Wild-type (WT) cells harboring wild-type or mutated *P*_*ATG39*_*-GFP* reporters were grown at 25 °C until exponential phase and treated with 3 μg/ml tunicamycin (TM) (C) or incubated under nitrogen-starved conditions (**D**) for the indicated time. The *GFP* mRNA levels were quantified by qRT-PCR analysis, and relative mRNA levels were calculated using *ACT1* mRNA. The values are plotted as the fold change from wild-type cells harboring the integration which expresses GFP under the control of wild-type *ATG39* promoter at the time of TM addition or nitrogen removal. The data show mean ± SEM (n = 3). **P < 0.01 as determined by Student’s *t*-test.

Msn2/4 activate transcription of stress responsive genes by binding to the STRE element, 5′-CCCCT-3′, located in the promoter of their target genes^26,27^. Our analysis using JASPAR, a database of transcription factor binding profiles (http://jaspar.genereg.net/), revealed that two putative STRE elements exist in the *ATG39* promoter (Fig. 3A). To examine whether the STRE elements are required for Msn2/4 to upregulate *ATG39* transcription, we mutated them in the *P*_*ATG39*_*-GFP* reporter. Msn4 overexpression failed to increase *GFP* expression from the *P*_*ATG39*_*-GFP* reporter mutated in the STRE elements (Fig. 3B). We next asked whether the STRE elements are important for activation of the *ATG39* promoter in response to ER stress. Activation of the *ATG39* promoter was diminished by either of the mutations in two STRE elements (Supplementary Fig. 4A). They synergistically reduced *ATG39* promoter activity during ER stress response (Fig. 3C and Supplementary Fig. 4A). Similar results were obtained when the *ATG39* promoter was activated by nitrogen starvation (Fig. 3D and Supplementary Fig. 4B). These results suggest that Msn2/4 positively regulate *ATG39* expression through the STRE elements located in its promoter.

### Msn2/4 positively regulate ER-phagy

**Figure 4:**
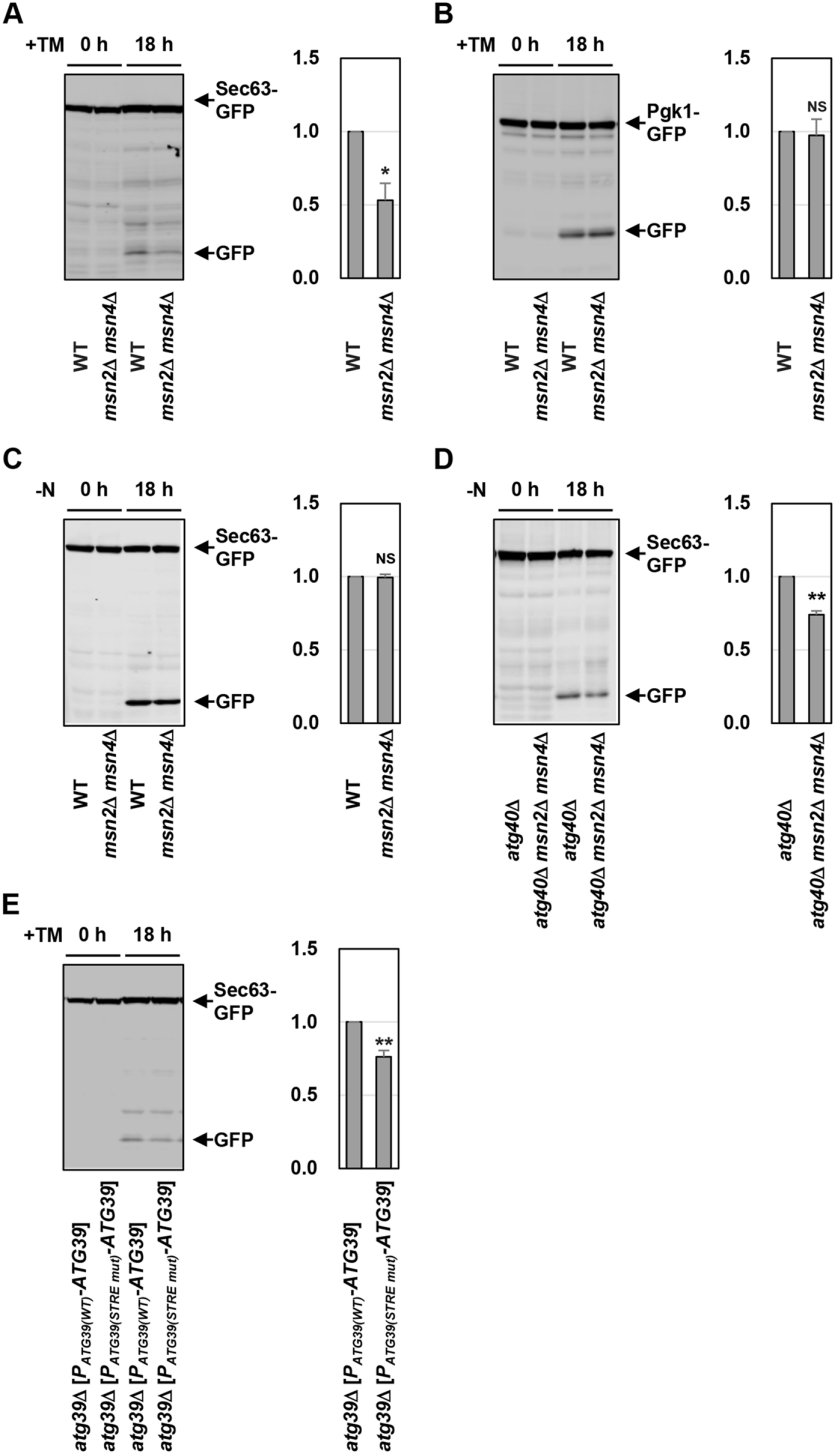
Msn2/4 positively regulate ER-phagy. (**A**) Sec63-GFP degradation in ER-stressed *msn2 msn4* mutant. Wild-type (WT) and *msn2 msn4* mutant strains harboring GFP-tagged *SEC63* were grown at 25 °C until exponential phase and treated with 3 μg/ml tunicamycin (TM) for 18 h. Extracts prepared from each cell were immunoblotted with anti-GFP antibodies. The intensities of free GFP were measured and normalized to the Sec63-GFP level. The values are plotted as the fold change from wild-type cells. The data show mean ± SEM (n = 3). *P < 0.01 as determined by Student’s *t*-test. (**B**) Pgk1-GFP degradation in ER-stressed *msn2 msn4* mutant. Wild-type (WT) and *msn2 msn4* mutant strains harboring GFP-tagged *PGK1* were grown at 25 °C until exponential phase and treated with 3 μg/ml tunicamycin (TM) for 18 h. Extracts prepared from each cell were immunoblotted with anti-GFP antibodies. The intensities of free GFP were measured and normalized to the Pgk1-GFP level. The values are plotted as the fold change from wild-type cells. The data show mean ± SEM (n = 3). NS, not statistically significant (*P* > 0.05), as determined by Student’s *t*-test. (**C**) Sec63-GFP degradation in nitrogen-starved *msn2 msn4* mutant. Wild-type (WT) and *msn2 msn4* mutant strains harboring GFP-tagged *SEC63* were grown at 25 °C until exponential phase and incubated under nitrogen-starved conditions for 18 h. Extracts prepared from each cell were immunoblotted with anti-GFP antibodies. The intensities of free GFP were measured and normalized to the Sec63-GFP level. The values are plotted as the fold change from wild-type cells. The data show mean ± SEM (n = 3). NS, not statistically significant (*P* > 0.05), as determined by Student’s *t*-test. (**D**) Effects of *msn2 msn4* mutations on Sec63-GFP degradation in the *atg40* mutant cells. Indicated mutant strains harboring GFP-tagged *SEC63* were grown at 25 °C until exponential phase and incubated under nitrogen-starved conditions for 18 h. Extracts prepared from each cell were immunoblotted with anti-GFP antibodies. The intensities of free GFP were measured and normalized to the Sec63-GFP level. The values are plotted as the fold change from wild-type cells. The data show mean ± SEM (n = 4). **P < 0.01 as determined by Student’s *t*-test. (**E**) Sec63-GFP degradation in cells expressing *ATG39* under the control of mutated *ATG39* promoter. The *atg39* mutant strains harboring GFP-tagged *SEC63* and the integration which expresses *ATG39* under the control of wild-type or mutated *ATG39* promoter were grown at 25 °C until exponential phase and treated with 3 μg/ml tunicamycin (TM) for 18 h. Extracts prepared from each cell were immunoblotted with anti-GFP antibodies. The intensities of free GFP were measured and normalized to the Sec63-GFP level. The values are plotted as the fold change from cells harboring the wild-type *ATG39* integration. The data show mean ± SEM (n = 4). **P < 0.01 as determined by Student’s *t*-test.

Next, we investigated whether *ATG39* induction mediated by Msn2/4 is important for activation of ER-phagy. We previously revealed that Atg39 acts as a major ER-phagy receptor in ER stress response^9^: diminished *ATG39* induction in response to ER stress reduces degradation of Sec63 ER transmembrane protein. Therefore, we investigated the role of Msn2/4 in ER stress-induced ER-phagy using the strain which expresses the carboxyl-terminally GFP-tagged Sec63 (Sec63-GFP)^6,9^. GFP is resistant to the vacuole-resident proteases, and thus autophagic degradation of GFP-tagged protein yields free GFP^28^. When wild-type cells expressing Sec63-GFP were treated with tunicamycin, free GFP production from Sec63-GFP was observed^9^ (Fig. 4A). Compared with wild-type cells, free GFP was decreased in the *msn2 msn4* double mutant cells (Fig. 4A). This result suggests that Msn2/4 are involved in autophagic degradation of Sec63-GFP caused by ER stress. We next examined whether *msn2 msn4* double mutations generally reduce autophagic activities during ER stress response. We monitored non-selective autophagy using strains that express the cytoplasmic Pgk1 tagged by GFP (Pgk1-GFP)^29^. Autophagic degradation of Pgk1-GFP in the *msn2 msn4* double mutant cells was comparable to that in wild-type cells (Fig. 4B). This result suggests that Msn2/4 are dispensable for activation of non-selective autophagy during ER stress response.

We next examined whether Msn2/4 are involved in nitrogen starvation-induced ER-phagy. Nitrogen starvation caused Sec63-GFP degradation in the *msn2 msn4* double mutant cells at a level comparable to that in wild-type cells (Fig. 4C). This result seems to be consistent with our previous observation that degradation of Sec63-GFP in response to nitrogen starvation is unaffected by the *atg39* single mutation^9^. We previously revealed that Sec63-GFP degradation in response to nitrogen starvation is partly blocked by *atg40* single mutation, and this *atg40* phenotype is enhanced by *atg39* mutation^9^. Accordingly, the inhibitory effect of *msn2 msn4* double mutations on nitrogen starvation-induced ER-phagy might be detected only in an *atg40* mutant background. Alternatively, reduced *ATG39* induction in the *msn2 msn4* double mutant cells might be enough to activate Atg39-dependent ER-phagy normally, since *ATG39* induction caused by nitrogen starvation is significantly greater than that caused by ER stress (Fig. 2A–C). Therefore, we compared degradation of Sec63-GFP in response to nitrogen starvation between the *atg40* single mutant and the *atg40 msn2 msn4* triple mutant cells. The *msn2 msn4* double mutations decreased Sec63-GFP degradation in nitrogen-starved *atg40* mutant cells (Fig. 4D). These results suggest that Msn2/4 are involved in ER-phagy induced by ER stress and nitrogen starvation.

We next investigated whether transcriptional activation of the *ATG39* gene mediated by Msn2/4 is important for regulation of ER-phagy. To test this, we generated strains expressing Atg39 under the control of the *ATG39* promoter mutated in the STRE elements. Sec63-GFP degradation in response to ER stress was decreased when Atg39 was expressed from the *ATG39* promoter mutated in the STRE elements (Fig. 4E). This result suggests that the control of *ATG39* promoter activity via the STRE elements is critical for regulation of ER-phagy.

### The PKA pathway is involved in regulation of ATG39 expression and ER-phagy

We previously revealed that Snf1 is involved in transcriptional activation of *ATG39* during ER stress response^9^. To examine the relationship between Msn2/4 and Snf1, we constructed the *msn2 msn4 snf1* triple mutant cells. *ATG39* induction after ER stress treatment was decreased by the *msn2 msn4 snf1* triple mutations compared with the *msn2 msn4* double mutations and the *snf1* single mutation (Supplementary Fig. 5). This result suggests that Msn2/4 and Snf1 independently regulate *ATG39* transcription.

**Figure 5:**
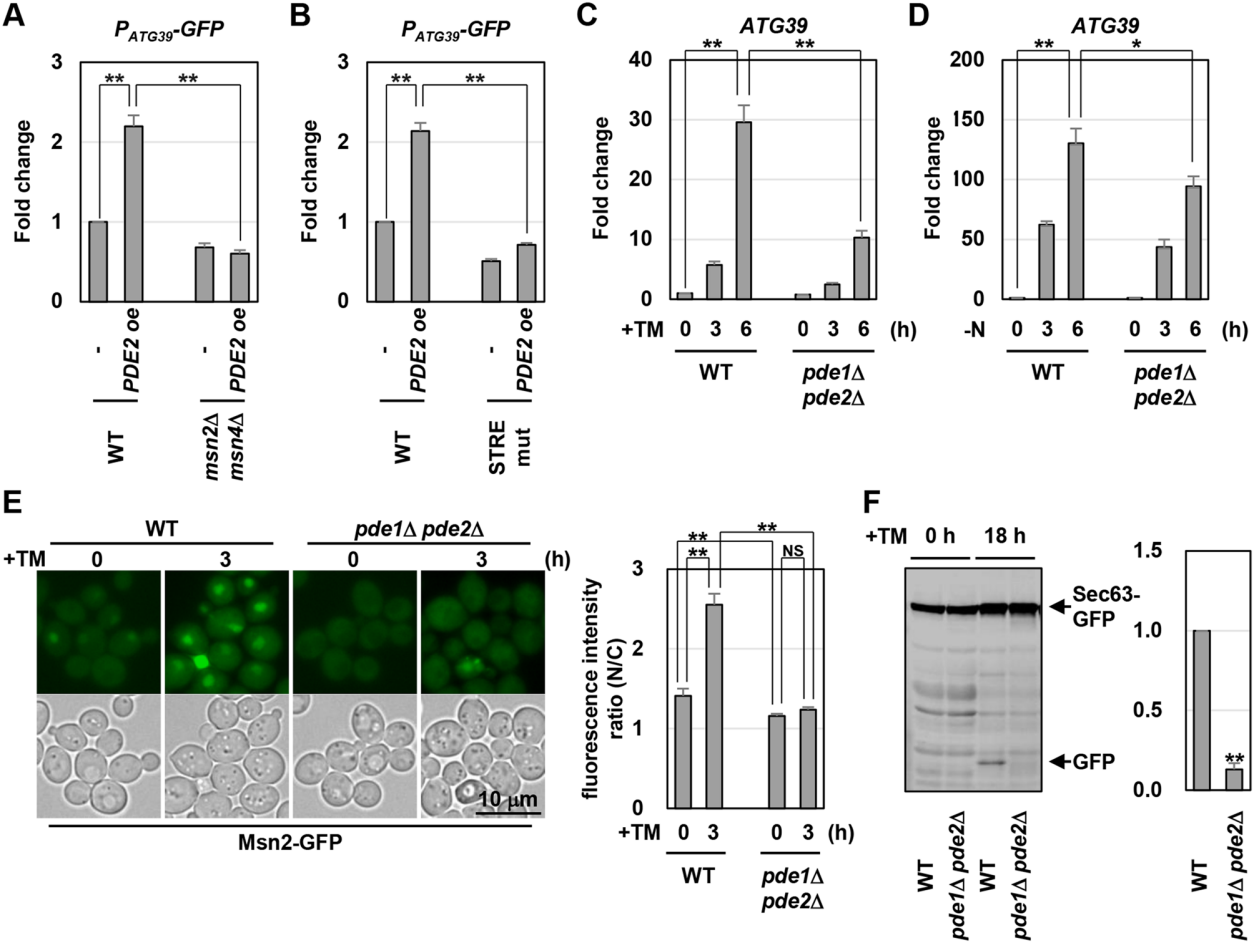
Pde1/2 positively regulate *ATG39* expression via Msn2/4. (**A**) Effects of *PDE2* overexpression on expression of the *P*_*ATG39*_*-GFP* reporter in the *msn2 msn4* mutant cells. Wild-type (WT) and *msn2 msn4* mutant strains harboring the *P*_*ATG39*_*-GFP* reporter and the plasmids with or without the *PDE2* genes were grown at 25 °C until exponential phase and then harvested. The *GFP* mRNA levels were quantified by qRT-PCR analysis, and relative mRNA levels were calculated using *ACT1* mRNA. The values are plotted as the fold change from wild-type cells harboring the empty plasmids. The data show mean ± SEM (n = 4). **P < 0.01 as determined by Student’s *t*-test. (**B**) Effects of *PDE2* overexpression on expression of mutated *P*_*ATG39*_*-GFP* reporter. Wild-type (WT) cells harboring wild-type or mutated *P*_*ATG39*_*-GFP* reporters and the plasmids with or without the *PDE2* genes were grown at 25 °C until exponential phase and then harvested. The *GFP* mRNA levels were quantified by qRT-PCR analysis, and relative mRNA levels were calculated using *ACT1* mRNA. The values are plotted as the fold change from cells harboring wild-type *P*_*ATG39*_*-GFP* reporter and the empty plasmids. The data show mean ± SEM (n = 4). **P < 0.01 as determined by Student’s *t*-test. (**C**,**D**) The *ATG39* mRNA levels in *pde1 pde2* mutant. Wild-type (WT) and *pde1 pde2* mutant strains were grown at 25 °C until exponential phase and treated with 3 μg/ml tunicamycin (TM) (**C**) or incubated under nitrogen-starved conditions (**D**) for the indicated time. The *ATG39* mRNA levels were quantified by qRT-PCR analysis, and relative mRNA levels were calculated using *ACT1* mRNA. The values are plotted as the fold change from wild-type cells at the time of TM addition or nitrogen removal. The data show mean ± SEM (n > 3). *P < 0.05 and **P < 0.01 as determined by Student’s *t*-test. (**E**) Cellular localization of Msn2. Wild-type (WT) and *pde1 pde2* mutant strains harboring GFP-tagged *MSN2* were grown at 25 °C until exponential phase and treated with 3 μg/ml tunicamycin (TM) for 3 h, and subjected to microscopy. The fluorescence intensities were measured, and then the ratios (N/C) of the fluorescence intensity per unit area in the nucleus/that in the cytoplasm were calculated. The graphs show mean ± SEM (n = 30). **P < 0.01 as determined by Student’s *t*-test. NS, not statistically significant (*P* > 0.05). Scale bar, 10 μm. (**F**) Sec63-GFP degradation in ER-stressed *pde1 pde2* mutant. Wild-type (WT) and *pde1 pde2* mutant strains harboring GFP-tagged *SEC63* were grown at 25 °C until exponential phase and treated with 3 μg/ml tunicamycin (TM) for 18 h. Extracts prepared from each cell were immunoblotted with anti-GFP antibodies. The intensities of free GFP were measured and normalized to the Sec63-GFP level. The values are plotted as the fold change from wild-type cells. The data show mean ± SEM (n = 3). *P < 0.01 as determined by Student’s *t*-test.

Here, we isolated the multicopy plasmid carrying the *PDE2* gene by the genetic screen. Pde2 is a cyclic AMP (cAMP) phosphodiesterase and downregulates the PKA activity by decreasing the level of cAMP^18,22^. It is well-known that PKA negatively regulates Msn2/4^18,24,25^. These previous findings raised the possibility that Pde2 positively regulates *ATG39* expression and ER-phagy upstream of Msn2/4. We first examined the effect of Pde2 overexpression on growth of cells harboring *P*_*ATG39*_*-HIS3*. Introduction of a multicopy plasmid expressing Pde2 facilitated growth of the reporter cell on SD-His + AT (Supplementary Fig. 6A). We also found that mRNAs from endogenous *ATG39* gene and the *P*_*ATG39*_*-GFP* reporter were increased by introduction of a multicopy plasmid expressing Pde2 (Fig. 5A and Supplementary Fig. 6B). These results suggest that *ATG39* promoter activity is upregulated by Pde2 overexpression. Next, we examined whether *ATG39* upregulation caused by Pde2 overexpression depends on the function of Msn2/4. In the *msn2 msn4* double mutant cells, Pde2 overexpression failed to increase *GFP* expression from the *P*_*ATG39*_*-GFP* reporter (Fig. 5A). Furthermore, mutations of the STRE elements in the *P*_*ATG39*_*-GFP* reporter significantly inhibited *GFP* upregulation caused by Pde2 overexpression (Fig. 5B). These results suggest that Msn2/4 are required for Pde2 to upregulate *ATG39* promoter activity. We next investigated whether Pde2 is involved in *ATG39* induction in response to ER stress and nitrogen starvation. The budding yeast possesses another cAMP phosphodiesterase which is encoded by the *PDE1* gene^30^, and Pde1 functions redundantly with Pde2 to regulate the cAMP level^18,30,31^. Therefore, we constructed the *pde1 pde2* double mutant cells. Similar to *msn2 msn4* double mutations, *pde1 pde2* double mutations partly but significantly diminished *ATG39* induction caused by ER stress and nitrogen starvation (Fig. 5C,D). These results suggest that Pde1/2 positively regulate *ATG39* expression under ER-stressed and nitrogen-starved conditions.

PKA negatively regulates Msn2/4 by promoting their nuclear export^18,25^. Previous studies have already revealed that Msn2 accumulates in the nucleus in response to nitrogen starvation and its nuclear accumulation is clearly inhibited by hyperactivation of PKA^32,33^. Additionally, it has been reported that ER stress leads to inactivation of PKA^34^. Therefore, we examined whether ER stress causes nuclear accumulation of Msn2 using the strains which express the carboxyl-terminally GFP-tagged Msn2 (Msn2-GFP). Under normal conditions, Msn2-GFP distributed throughout the cytoplasm and nucleus (Fig. 5E). Msn2-GFP accumulated in the nucleus after ER stress treatment. However, nuclear accumulation of Msn2-GFP induced by ER stress was clearly inhibited in the *pde1 pde2* double mutant cells. We also found that nuclear accumulation of Msn2 is caused by nitrogen starvation and inhibited by *pde1 pde2* double mutations (Supplementary Fig. 6C). These results suggest that the PKA pathway negatively regulates *ATG39* transcription by promoting the nuclear export of Msn2/4.

Finally, we investigated whether Pde1/2 are involved in regulation of ER-phagy. Sec63-GFP degradation in response to ER stress was modestly diminished in the *pde1 pde2* double mutant cells (Fig. 5F), suggesting that Pde1/2 positively regulate ER stress-induced ER-phagy. We also monitored non-selective autophagy in the *pde1 pde2* double mutant cells. Pgk1-GFP degradation in response to ER stress was significantly reduced by *pde1 pde2* double mutations (Supplementary Fig. 6D). This result is consistent with the previous findings that non-selective autophagy is inhibited by activation of PKA^33,35^. These results suggest that the PKA pathway not only regulates *ATG39* transcription via Msn2/4 but also influences general autophagic activities.

## Discussion

**Figure 6:**
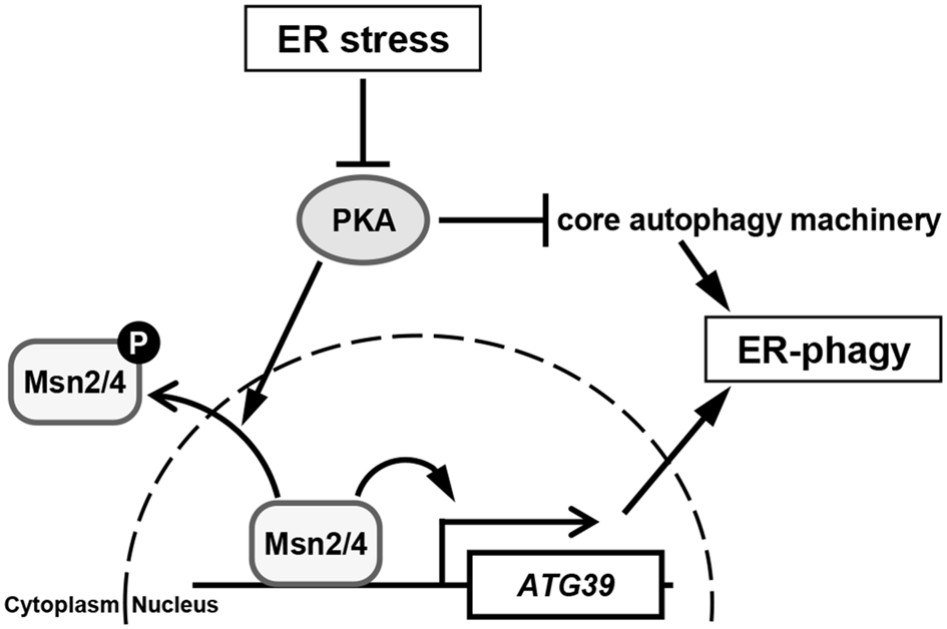
Proposed model for Msn2/4 in ER stress-induced ER-phagy.

In this study, we identified two closely related transcriptional activators, Msn2 and Msn4, as a positive regulator of *ATG39* transcription. We also found that the *ATG39* promoter contains two Msn2/4-binding consensus sequences termed STRE, whose disruption downregulates *ATG39* promoter activity to a similar extent as *msn2 msn4* double mutations. These results suggest that *ATG39* expression is transcriptionally activated via the STRE elements by Msn2 and Msn4 (Fig. 6). We also isolated Pde2 cAMP phosphodiesterase as a positive regulator of *ATG39* transcription. Pde2 functions redundantly with Pde1 to downregulate the PKA activity^18^. PKA negatively regulates Msn2 and Msn4 by promoting their nuclear export^18,25^. Thus, Pde1 and Pde2 positively regulate Msn2 and Msn4. Consistently, the *pde1 pde2* double mutants exhibited the phenotypes similar to that observed in the *msn2 msn4* double mutants: *ATG39* induction in response to ER stress and nitrogen starvation was diminished in the *pde1 pde2* double mutant cells. We also found that *ATG39* promoter activity could not be activated by Pde2 overexpression when the *MSN2* and *MSN4* genes were deleted or the STRE elements were mutated. Furthermore, nuclear accumulation of Msn2 in response to ER stress and nitrogen starvation was clearly inhibited by *pde1 pde2* double mutations. These results suggest that Pde1/2 positively regulate Msn2/4-mediated *ATG39* transcription probably through PKA. Additionally, it is suggested that PKA regulates autophagy through both Msn2/4-dependent and -independent mechanisms from the following observations: (1) *pde1 pde2* double mutations inhibited autophagic degradation of the Sec63 ER transmembrane protein more severely than *msn2 msn4* double mutations (Figs. 4A, 5F); (2) *pde1 pde2* double mutations inhibited autophagic degradation of the Pgk1 cytoplasmic protein, but *msn2 msn4* double mutations did not (Fig. 4B and Supplementary Fig. 6D). It has been reported that PKA directly phosphorylates and negatively regulates Atg1, a kinase essential for activation of autophagy^1,2,36^. Thus, the PKA pathway not only regulates ER-phagy by controlling Msn2/4-mediated *ATG39* transcription but also modulates the activity of the core autophagy-related machinery (Fig. 6).

A previous study showed that ER stress downregulates the PKA activity^34^. Here, we observed nuclear accumulation of Msn2 in ER-stressed cells. These findings suggest that ER stress inactivates PKA and consequently promotes Msn2 nuclear accumulation to induce *ATG39* transcription. Nuclear accumulation of Msn2 under nitrogen-starved conditions has been observed^32,33^. However, there is little compelling evidence that the PKA activity is controlled by nitrogen availability. It is well-known that nitrogen starvation leads to inactivation of target of rapamycin complex 1 (TORC1)^37,38^. A previous study revealed that TORC1 promotes nuclear export of Msn2^39^. Therefore, it is possible that during nitrogen starvation, Msn2 nuclear accumulation and consequent *ATG39* transcriptional induction are mediated by inactivation of TORC1, rather than inactivation of PKA.

*ATG39* transcription was partly activated by ER stress and nitrogen starvation even when the *MSN2* and *MSN4* genes were deleted or the STRE elements were mutated. We previously revealed that *ATG39* transcription is repressed by Mig1 and Mig2 transcription factors and this repression itself is negatively regulated by Snf1 AMPK^9^. We found here that unlike *snf1* mutation, *msn2 msn4* double mutations diminished *ATG39* induction in response to nitrogen starvation. Furthermore, *snf1* mutation downregulated *ATG39* induction caused by ER stress in the *msn2 msn4* double mutant cells. These results suggest that Msn2/4 regulate *ATG39* transcription in a manner independent of Snf1. Additionally, our findings that *ATG39* induction in response to ER stress was still observed in the *snf1 msn2 msn4* triple mutant cells predicted the existence of unidentified activators for *ATG39* expression. Here, we developed a genetic screen to identify the positive regulator of *ATG39* transcription. However, we could not isolate any candidates except Msn2/4 transcription factors and negative regulators of the PKA pathway such as Pde2 and Gpb1. All factors we identified here upregulate *ATG39* transcription through the STRE elements located in its promoter. Therefore, it is expected that unidentified positive regulators may be further isolated using a reporter in which the STRE elements are mutated. Such investigation would enhance our understandings of transcriptional regulation of autophagy receptors and its importance in regulation of the activity of selective autophagy.

## Methods

### Plasmids

The YCplac33-P_ATG39_-GFP, YCplac33-P_MCM2_-GFP and YCplac33-P_ATG39_-ATG39 plasmids were described previously^9^. The *P*_*ATG39*_*-HIS3* construct was generated as follows. A 990-bp genomic fragment containing the *ATG39* promoter was amplified from the YCplac33-P_ATG39_-GFP plasmid by PCR with the following primers: 5′-CTCTAGAGGATCCCCGGGAAAAACTGTGCTCCTAGCAG-3′ and 5′-GGCTGGTTCTGCCATTTTAGGTCCGACAACTCG-3′. A DNA fragment encoding *HIS3MX6* followed by the *ADH1* terminator (*T*_*ADH1*_) was amplified from the pFA6a-GFP-HIS3MX6 vector by PCR with the following primers: 5′-ATGGCAGAACCAGCCCAAAAAAAGC-3′ and 5′-TCGAGCTCGGTACCCGGGCAGTATAGCGACCAGCATTCACATACG-3′. The amplified 5′ upstream sequences of the *ATG39* gene, together with the *HIS3MX6-T*_*ADH1*_ DNA fragment, were fused to the YCplac33 vector by In-Fusion cloning kits (Takara), yielding the YCplac33-P_ATG39_-HIS3 plasmid. Similarly, the YCplac33-P_MCM2_-HIS3 plasmid was constructed. The primers used to amplify *P*_*MCM2*_ are 5′-CTCTAGAGGATCCCCGGGAAAGGCGTCTATCCACTTGC-3′ and 5′-GGCTGGTTCTGCCATCACTTATATTGTAGTTGTG-3′. The *MSN2*, *MSN4* and *PDE2* genes were amplified from the *Saccharomyces cerevisiae* W303 derivative by PCR with the following primers: 5′-CTCTAGAGGATCCCCGGGGTTTCCAGCGAAAGAGACAG-3′ and 5′-TCGAGCTCGGTACCCGGGTAAAAGTAGCAAACTGGTAG-3′ for *MSN2*; 5′-CTCTAGAGGATCCCCGGGAAACCCGAGCTAGAACTAGG-3′ and 5′-TCGAGCTCGGTACCCGGGAGCAAACGTCGTACCAATCC-3′ for *MSN4*; 5′-CTCTAGAGGATCCCCGGGATTATCTTTGTAATCTGCAG-3′ and 5′-TCGAGCTCGGTACCCGGGCTTTGCATATACCAACACAG-3′ for *PDE2*. The amplified *MSN2*, *MSN4* and *PDE2* genes were fused to the YEplac181 vector by In-Fusion cloning kits (Takara), yielding the YEplac181-MSN2, YEplac181-MSN4 and YEplac181-PDE2 plasmids, respectively. Mutations of STREs in the *ATG39* promoter were generated by oligonucleotide-directed PCR using the following primers: 5′-CCTCCGGACCATCTACCCCGGTGTG-3′ and 5′-TAGATGGTCCGGAGGGTTAACTGTC-3′ for STRE1 mutation; 5′-GCGCAAAGATGTTACCGCAAAATGG-3′ and 5′-GTAACATCTTTGCGCGACAGCTGC-3′ for STRE2 mutation. To generate the *P*_*ATG39(STREmut)*_*-GFP* and *P*_*ATG39(STREmut)*_*-ATG39* integrations, the inserts in the YCplac33 plasmids were subcloned into the pRS306 vector. Plasmids used in this study are described in Supplementary Table 2.

### Strains

Standard procedures were followed for yeast manipulations^40^. SD(–N) medium (0.17% (w/v) yeast nitrogen base without amino acids and ammonium sulfate and 2% (w/v) glucose) was used to induce nitrogen starvation. Yeast strains harboring the complete gene deletions (*MSN2*, *MSN4*, *PDE1* and *PDE2*) and carboxyl-terminally GFP-tagged *MSN2* were generated by a PCR-based method as described previously^41^. Primer sets were designed such that 46 bases at the 5′ end of primers were complementary to those at the corresponding region of the target gene, and 20 bases at their 3′ end were complementary to the pFA6a sequence, 5′-TGCAGTACTCTGCGGGTGTATACAG-3′ or 5′-ATTTGACTGTATTACCAATGTCAGC-3′. All strains produced by a PCR-based method were verified by colony PCR amplification to confirm that replacement had occurred at the expected locus. Strains carrying the *P*_*ATG39(STREmut)*_*-GFP* and *P*_*ATG39(STREmut)*_*-ATG39* integrations were constructed by integrating the linearized pRS306-P_ATG39(STREmut)_-GFP and pRS306-P_ATG39(STREmut)_-ATG39 plasmids, respectively. Strains used in this study are listed in Supplementary Table 3.

### Identification of genes activating the *P*_*ATG39*_*-HIS3* reporter

Wild-type cells harboring the *P*_*ATG39*_*-HIS3* reporter poorly grew on synthetic defined media that lack histidine and contain 3-amino-1*H*-1,2,4-triazole (AT) due to low expression of *HIS3MX6*. To identify genes whose overexpression allows cells harboring the *P*_*ATG39*_*-HIS3* reporter to form colonies on synthetic defined media lacking histidine, the multicopy genomic libraries were screened as follows. A *Saccharomyces cerevisiae* YEp13 genomic library was transformed into cells harboring the *P*_*ATG39*_*-HIS3* reporter. Cells were plated onto synthetic defined media lacking leucine and allowed to grow by incubation at 30 °C for 2 days. Transformants grown on synthetic defined media lacking leucine were transferred on synthetic defined media lacking histidine and containing AT and allowed to grow by incubation at 30 °C. Plasmids were collected from colonies that grew on selective media, and the ends of genomic inserts were sequenced.

### RNA isolation and RT-PCR

Preparation of total RNA and generation of cDNA were performed as described previously^19^. The cDNAs were quantitated by a quantitative real-time RT-PCR (qRT-PCR) method using QuantStudio 5 real-time PCR systems (Applied Biosystems) with TB Green Premix Ex Taq (Takara), and levels of gene expression were normalized to *ACT1* expression. Primers used to analyze the mRNA level are described in Supplementary Table 4.

### Protein extraction, western blot analysis and antibodies

Preparation of protein extracts and Western blot analysis were performed as described previously^19^. Anti-GFP antibody from mouse IgG1κ (clones 7.1 and 13.1) (Roche) was used. Detection and quantification were carried out by using the Odyssey Imaging Systems (LI-COR Biosciences). Statistical analysis was performed with Excel (Microsoft).

### Microscopy

To visualize GFP-tagged Msn2 in living cells, cells were grown at 25 °C until exponential phase and treated with 3 μg/ml tunicamycin (TM) for 3 h. Cells were then harvested, suspended in SD medium, and observed immediately using a Keyence BZ-X700 microscope (Keyence) with a PlanAproλ 100 × NA 1.45 oil objective lens (Nikon). Fluorescence intensities were quantified using Hybrid Cell Count BZ-H2C software (Keyence).

## Supplementary Information

Supplementary Information.
