# Supplementary material for: Msn2/4 transcription factors positively regulate expression of Atg39 ER-phagy receptor
Source: Sci Rep. 2021 Jun 7;11:11919. doi: 10.1038/s41598-021-91480-0 (PMC8184937; doi:10.1038/s41598-021-91480-0)
Supplement: Supplementary file 1 — Supplementary Information. [file 41598_2021_91480_MOESM1_ESM.pdf]

Title:

Msn2/4 transcription factors positively regulate expression of Atg39 ER-phagy receptor

Authors:

Tomoaki Mizuno* and Kenji Irie

Affiliation:

Department of Molecular Cell Biology, Faculty of Medicine, University of Tsukuba, Tsukuba, Japan.

*Correspondence and requests for materials should be addressed to T. M. (mizuno@md.tsukuba.ac.jp)

Supplementary Figure 1.

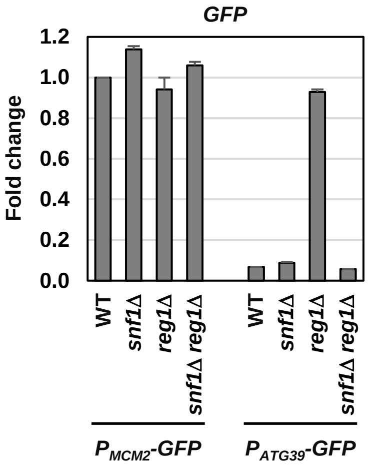

Supplementary Figure 1. The *P_{ATG39}-GFP* reporter activity is upregulated by Snf1 activation. Wild-type (WT) and indicated mutant strains harboring the *P_{ATG39}-GFP* reporter were grown at 25 ºC until exponential phase and then harvested. The *GFP* mRNA levels were quantified by qRT-PCR analysis, and relative mRNA levels were calculated using *ACT1* mRNA. The values are plotted as the fold change from wild-type cells. The data show mean ± SEM (n = 3).

Supplementary Figure 2.

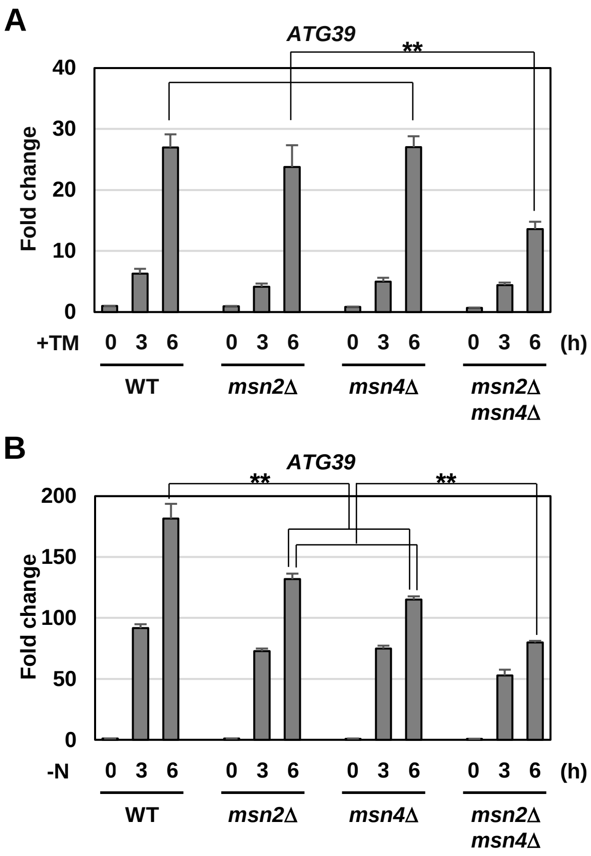

Supplementary Figure 2. Msn2 and Msn4 redundantly regulate *ATG39* expression.

(A, B) Wild-type (WT) and indicated mutant strains were grown at 25 ^{o}C until exponential phase and treated with 3 μg/ml tunicamycin (TM) (A) or incubated under nitrogen-starved conditions (B) for the indicated time. The *ATG39* mRNA levels were quantified by qRT-PCR analysis, and relative mRNA levels were calculated using *ACT1* mRNA. The values are plotted as the fold change from wild-type cells at the time of TM addition or nitrogen removal. The data show mean ± SEM (n = 3). NS, not statistically significant ($P > 0.05$), as determined by Student's *t*-test.

Supplementary Figure 3.

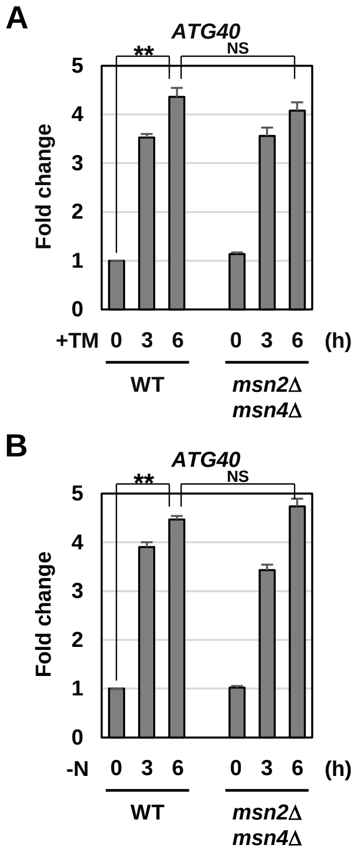

Supplementary Figure 3. The *ATG40* mRNA levels were unchanged in *msn2 msn4* mutants. (A, B) Wild-type (WT) and *msn2 msn4* mutant strains were grown at 25 ^{o}C until exponential phase and treated with 3 μg/ml tunicamycin (TM) (A) or incubated under nitrogen-starved conditions (B) for the indicated time. The *ATG40* mRNA levels were quantified by qRT-PCR analysis, and relative mRNA levels were calculated using *ACT1* mRNA. The values are plotted as the fold change from wild-type cells at the time of TM addition or nitrogen removal. The data show mean $\pm$ SEM (n = 3). NS, not statistically significant ($P > 0.05$), as determined by Student's *t*-test.

Supplementary Figure 4.

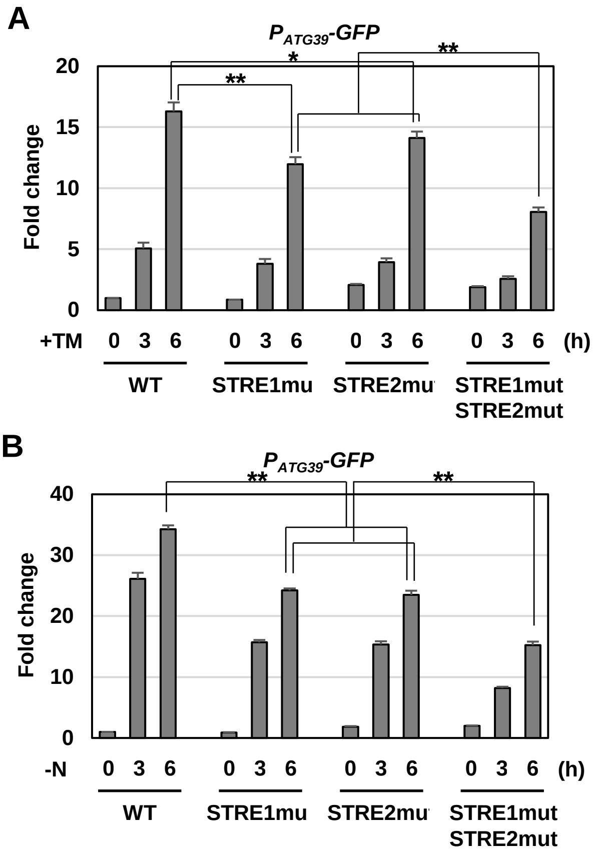

Supplementary Figure 4. The STRE elements redundantly regulate *ATG39* expression.

(A, B) Wild-type (WT) cells harboring wild-type or mutated P_{ATG39}-*GFP* reporters were grown at 25 ºC until exponential phase and treated with 3 μg/ml tunicamycin (TM) (A) or incubated under nitrogen-starved conditions (B) for the indicated time. The *GFP* mRNA levels were quantified by qRT-PCR analysis, and relative mRNA levels were calculated using *ACT1* mRNA. The values are plotted as the fold change from wild-type cells at the time of TM addition or nitrogen removal. The data show mean ± SEM (n > 3). **P* < 0.05 and ***P* < 0.01 as determined by Student's *t*-test. NS, not statistically significant (*P* > 0.05).

Supplementary Figure 5.

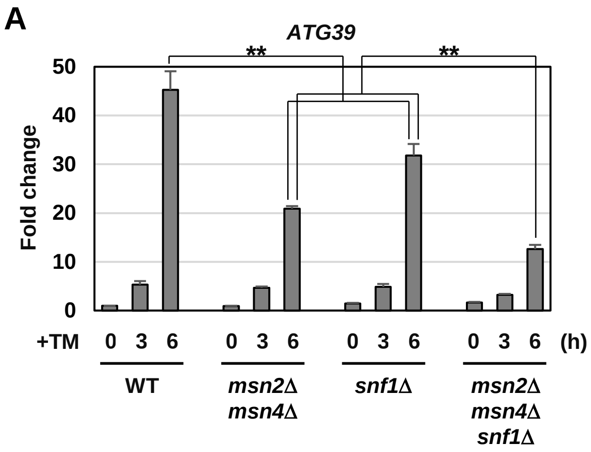

Supplementary Figure 5. Msn2/4 and Snf1 independently regulate *ATG39* expression.

The *ATG39* mRNA levels in *msn2 msn4 snf1* mutant. Wild-type (WT) and indicated mutant strains were grown at 25 °C until exponential phase and treated with 3 μg/ml tunicamycin (TM) for the indicated time. The *ATG39* mRNA levels were quantified by qRT-PCR analysis, and relative mRNA levels were calculated using *ACT1* mRNA. The values are plotted as the fold change from wild-type cells at the time of TM addition. The data show mean ± SEM (n = 4). ***P* < 0.01 as determined by Student's *t*-test.

Supplementary Figure 6.

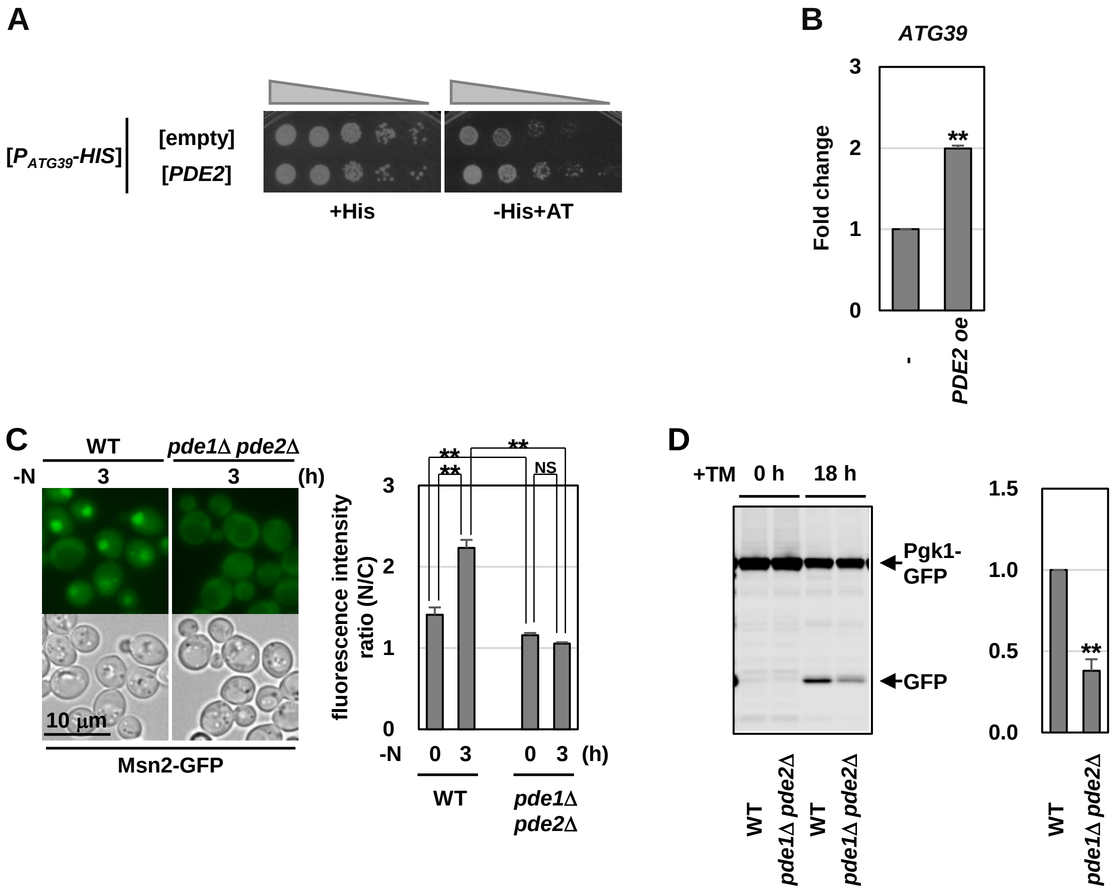

Supplementary Figure 6. The cAMP phosphodiesterases regulate *ATG39* expression and the activity of non-selective autophagy.

(A) Effects of *PDE2* overexpression on growth of cells harboring P_{ATG39}-*HIS3* reporter. Wild-type (WT) cells harboring the P_{ATG39}-*HIS3* reporter and the plasmids with or without the *PDE2* gene were spotted onto synthetic defined media lacking or containing histidine (His) and 3-amino-1H-1,2,4-triazole (AT), and incubated at 25 ^{o}C.

(B) Effects of *PDE2* overexpression on *ATG39* expression. Wild-type strains harboring the plasmids with or without the *PDE2* genes were grown at 25 ^{o}C until exponential phase and then harvested. The *ATG39* mRNA levels were quantified by qRT-PCR analysis, and relative mRNA levels were calculated using *ACT1* mRNA. The values are plotted as the fold change from wild-type cells harboring the empty plasmids. The data show mean ± SEM (n = 4). **P < 0.01 as determined by Student's *t*-test.

(C) Cellular localization of Msn2. Wild-type (WT) and *pde1 pde2* mutant strains harboring GFP-tagged *MSN2* were grown at 25 ^{o}C until exponential phase, incubated under nitrogen-starved conditions for 3 hr, and subjected to microscopy. The fluorescence intensities were measured, and then the ratios (N/C) of the fluorescence intensity per unit area in the nucleus/that in the cytoplasm were calculated. The graphs show mean ± SEM (n = 30). **P < 0.01 as determined by Student's *t*-test. NS, not statistically significant (P > 0.05). Scale bar, 10 μm.

(D) Pgk1-GFP degradation in ER-stressed *pde1 pde2* mutant. Wild-type (WT) and *pde1 pde2* mutant strains harboring GFP-tagged *PGK1* were grown at 25 ^{o}C until exponential phase and treated with 3 μg/ml tunicamycin (TM) for 18 hr. Extracts prepared from each cell were immunoblotted with anti-GFP antibodies. The intensities of free GFP were measured and normalized to the Pgk1-GFP level. The values are plotted as the fold change from wild-type cells. The data show mean ± SEM (n = 3). NS, not statistically significant (P > 0.05), as determined by Student's *t*-test.

Supplementary Figure 7
(Original data for Fig. 4A)

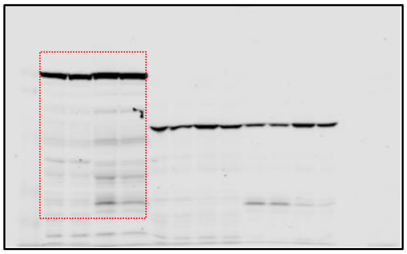

Supplementary Figure 8
(Original data for Fig. 4B)

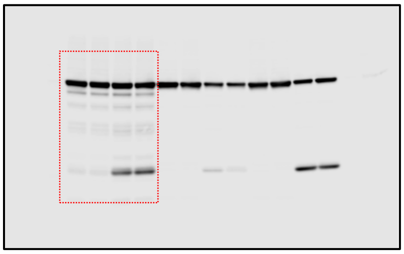

Supplementary Figure 9
(Original data for Fig. 4C)

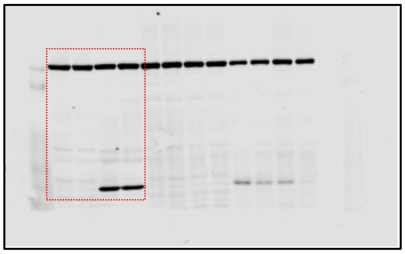

Supplementary Figure 10
(Original data for Fig. 4D)

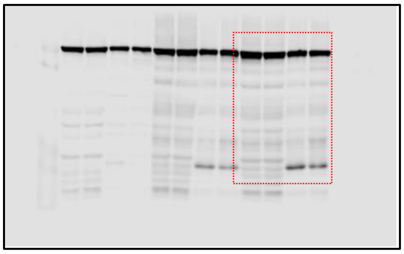

Supplementary Figure 11
(Original data for Fig. 4E)

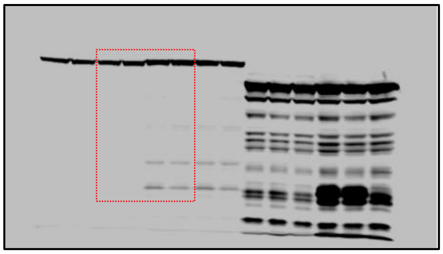

Supplementary Figure 12
(Original data for Fig. 5F)

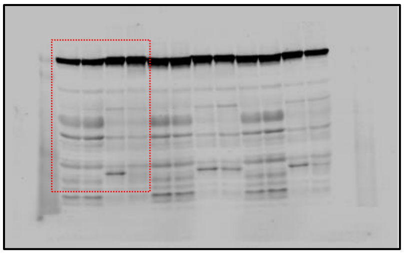

Supplementary Figure 13
(Original data for Supplementary Figure 6D)

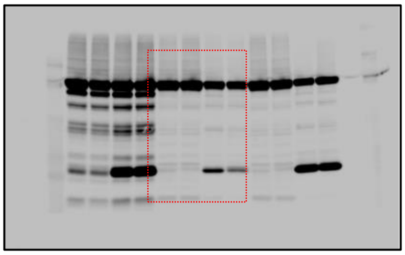

Supplementary Table 1

Group	Plasmid	Included genes
I	1	*NAB6, YML116W-A, ATR1, VAN1, TAF8*
I	2	*NAB6, YML116W-A, ATR1, VAN1, TAF8*
I	3	*NAB6, YML116W-A, ATR1, VAN1, TAF8*
I	4	*ATR1, VAN1*
I	5	*ATR1, VAN1*
I	6	*ATR1, VAN1*
I	7	*NDI1, YML119W, NGL3, NAB6, YML116W-A, ATR1, VAN1*
I	8	*ATR1, VAN1, TAF8*
II	9	*MIH1, MSN2, CCS1*
II	10	*MIH1, MSN2, CCS1*
II	11	*MIH1, MSN2, CCS1*
II	12	*MIH1, MSN2, CCS1*
III	13	*SNX3, HAP5, VTS1, PDE2*
III	14	*SNX3, HAP5, VTS1, PDE2*
III	15	*PDE2, PRT1, PRE10*
IV	16	*MRS6, GPB1*

Supplementary Table 1. Plasmids isolated by the genetic screen in this study.

Supplementary Table 2

Plasmids	Relevant markers	Source
pCgLEU2	*C. glabrata LEU2* in pUC19	Sakumoto, N. et al. 1999
pCgTRP1	*C. glabrata TRP1* in pUC19	Sakumoto, N. et al. 1999
pFA6a-kanMX6	*kanMX6*	Longtine, M. S. et al. 1998
pFA6a-HIS3MX6	*HIS3MX6*	Longtine, M. S. et al. 1998
pFA6a-natNT2	*natNT2*	Janke, C. et al. 2004
pFA6a-hphNT1	*hphNT1*	Janke, C. et al. 2004
pFA6a-GFP-HIS3MX6	*GFP-ADH 3'UTR-HIS3MX6*	Longtine, M. S. et al. 1998
YEplac181	*LEU2*	Gietz, R. D. & Sugino, A 1988
YCplac33	*URA3*	Gietz, R. D. & Sugino, A 1988
pRS306	*URA3*	Sikorski, R. S. & Hieter, P. 1989
YCplac33-P_{ATG39}-GFP	*URA3*, P_{ATG39}-*GFP*	Mizuno, T. et al. 2020
pRS306-P_{ATG39}-GFP	*URA3*, P_{ATG39}-*GFP*	Mizuno, T. et al. 2020
YEplac181-MSN2	*LEU2, MSN2*	this study
YEplac181-MSN4	*LEU2, MSN4*	this study
YEplac181-PDE2	*LEU2, PDE2*	this study
YCplac33-P_{ATG39}-HIS3	*URA3*, P_{ATG39}-*HIS3*	this study
YCplac33-P_{MCM2}-HIS3	*URA3*, P_{MCM2}-*HIS3*	this study
YCplac33-$P_{ATG39(STRE1mut)}$-GFP	*URA3*, $P_{ATG39(STRE1mut)}$-*GFP*	this study
YCplac33-$P_{ATG39(STRE2mut)}$-GFP	*URA3*, $P_{ATG39(STRE2mut)}$-*GFP*	this study
YCplac33-$P_{ATG39(STRE1mut\ STRE2mut)}$-GFP	*URA3*, $P_{ATG39(STRE1mut\ STRE2mut)}$-*GFP*	this study
YCplac33-$P_{ATG39(STRE1mut\ STRE2mut)}$-ATG39	*URA3*, $P_{ATG39(STRE1mut\ STRE2mut)}$-*ATG39*	this study
pRS306-$P_{ATG39(STRE1mut)}$-GFP	*URA3*, $P_{ATG39(STRE1mut)}$-*GFP*	this study
pRS306-$P_{ATG39(STRE2mut)}$-GFP	*URA3*, $P_{ATG39(STRE2mut)}$-*GFP*	this study
pRS306-$P_{ATG39(STRE1mut\ STRE2mut)}$-GFP	*URA3*, $P_{ATG39(STRE1mut\ STRE2mut)}$-*GFP*	this study
pRS306-$P_{ATG39(STRE1mut\ STRE2mut)}$-ATG39	*URA3*, $P_{ATG39(STRE1mut\ STRE2mut)}$-*ATG39*	this study

Supplementary Table 2. Plasmids used in this study.

Supplementary Table 3

Strains	Genotype	reference
10B	*MAT α ade2 trp1 can1 leu2 his3 ura3 GAL psi+ HOp-ADE2-HO 3' UTR*	Tadauchi, T. et al. 2001
10Ba	*MAT* **a** *ade2 trp1 can1 leu2 his3 ura3 GAL psi+ HOp-ADE2-HO 3' UTR*	Tadauchi, T. et al. 2001
10BD	*MAT* **a**/*MAT α ade2/ade2 trp1/trp1 can1/can1 leu2/leu2 his3/his3 ura3/ura3*	Tadauchi, T. et al. 2001
YCH101	*ade2 trp1 can1 leu2 his3 ura3 snf1 Δ::CgTRP1*	Mizuno, T. et al. 2015
YCH122	*ade2 trp1 can1 leu2 his3 ura3 reg1 Δ::CgLEU2*	Mizuno, T. et al. 2015
YCH124	*ade2 trp1 can1 leu2 his3 ura3 snf1 Δ::CgTRP1 reg1 Δ::CgLEU2*	Mizuno, T. et al. 2015
YCH301	*ade2 trp1 can1 leu2 his3 ura3 SEC63-GFP::HIS3MX6*	Mizuno, T. et al. 2020
YCH307	*ade2 trp1 can1 leu2 his3 ura3 SEC63-GFP::HIS3MX6 atg40 Δ::natNT2*	Mizuno, T. et al. 2020
YCH381	*ade2 trp1 can1 leu2 his3 ura3 PGK1-GFP::HIS3MX6*	Mizuno, T. et al. 2020
YCH401	*ade2 trp1 can1 leu2 his3 URA3::*P_{ATG39}*-GFP*	Mizuno, T. et al. 2020
YCH501	*ade2 trp1 can1 leu2 his3 URA3::*P_{ATG39}*-GFP msn2 Δ::kanMX6*	this study
YCH502	*ade2 trp1 can1 leu2 his3 URA3::*P_{ATG39}*-GFP msn4 Δ::CgTRP*	this study
YCH503	*ade2 trp1 can1 leu2 his3 URA3::*P_{ATG39}*-GFP msn2 Δ::kanMX6 msn4 Δ::CgTRP*	this study
YCH506	*ade2 trp1 can1 leu2 his3 URA3::*$P_{ATG39(STRE1mut)}$*-GFP*	this study
YCH507	*ade2 trp1 can1 leu2 his3 URA3::*$P_{ATG39(STRE2mut)}$*-GFP*	this study
YCH508	*ade2 trp1 can1 leu2 his3 URA3::*$P_{ATG39(STRE1mut\ STRE2mut)}$*-GFP*	this study
YCH511	*ade2 trp1 can1 leu2 his3 ura3 msn2 Δ::kanMX6 msn4 Δ::natNT2*	this study
YCH512	*ade2 trp1 can1 leu2 his3 ura3 snf1 Δ::CgTRP1 msn2 Δ::kanMX6 msn4 Δ::natNT2*	this study
YCH513	*ade2 trp1 can1 leu2 his3 ura3 pde1 Δ::CgLEU2 pde2 Δ::CgTRP1*	this study
YCH516	*ade2 trp1 can1 leu2 his3 ura3 SEC63-GFP::HIS3MX6 msn2 Δ::kanMX6 msn4 Δ::CgTRP*	this study
YCH517	*ade2 trp1 can1 leu2 his3 ura3 SEC63-GFP::HIS3MX6 atg40 Δ::natNT2 msn2 Δ::kanMX6 msn4 Δ::CgTRP*	this study
YCH518	*ade2 trp1 can1 leu2 his3 ura3 SEC63-GFP::HIS3MX6 pde1 Δ::CgLEU2 pde2 Δ::CgTRP1*	this study
YCH521	*ade2 trp1 can1 leu2 his3 ura3 PGK1-GFP::HIS3MX6 msn2 Δ::kanMX6 msn4 Δ::natNT2*	this study
YCH522	*ade2 trp1 can1 leu2 his3 ura3 PGK1-GFP::HIS3MX6 pde1 Δ::CgLEU2 pde2 Δ::CgTRP1*	this study
YCH526	*ade2 trp1 can1 leu2 his3 ura3 MSN2-GFP::HIS3MX6*	this study
YCH527	*ade2 trp1 can1 leu2 his3 ura3 MSN2-GFP::HIS3MX6 pde1 Δ::CgLEU2 pde2 Δ::CgTRP1*	this study
YCH531	*ade2 trp1 can1 leu2 his3 URA3::*P_{ATG39}*-ATG39 SEC63-GFP::HIS3MX6 atg39 Δ::kanMX6*	this study
YCH532	*ade2 trp1 can1 leu2 his3 URA3::*$P_{ATG39(STRE1mut\ STRE2mut)}$*-ATG39 SEC63-GFP::HIS3MX6 atg39 Δ::kanMX6*	this study

10B and YCH are W303 derivatives.

Supplementary Table 3. Strains used in this study.

Supplementary Table 4

Gene Name	Forw ard Primer	Reverse Primer
ACT1	TGCCGAAAGAATGCAAAAGG	TCTGGAGGAGCAATGATCTTGA
GFP	GGAGAGGGTGAAGGTGATGC	CTTCGGGCATGGCACTCTTG
ATG39	TCCTTTGCAGGAGAGGACGA	GTTCCGCCAACATTTGAGCC
ATG40	CAGTTGCCATTCCTTTGCAG	TGGGGACTGACCCAAAGAAG

Supplementary Table 4. Primers used to analyze the mRNA level in this study.
